# Efficient numerical treatment of time fractional advection diffusion equations for modeling heat, pollutant and particle transport using subdivision collocation

**DOI:** 10.1038/s41598-026-51148-z

**Published:** 2026-05-12

**Authors:** Saima Bibi, Syeda Tehmina Ejaz

**Affiliations:** Department of Mathematics, The Government Sadiq College Women University Bahawalpur, Bahawalpur, 63100 Pakistan

**Keywords:** Subdivision schemes, Fractional advection equation, Collocation algorithm, Stability, Engineering, Environmental sciences, Mathematics and computing

## Abstract

In numerical methods, fine mesh sizes are often necessary to obtain highly accurate solutions of the differential equations and this increases the memory consumption and decreases the efficiency of the calculation. This paper presents a subdivision collocation algorithm of time-fractional advection diffusion equation that is a model that is used to characterize anomalous diffusion in scientific and engineering systems. The fractional time derivative is discretized in the Caputo sense to model memory effects, whereas spatial approximation is done using subdivision schemes. The approach converts the problem into an effective and steady system of algebraic equations by collocating at spatial nodes. The consistency and error analysis indicate that the methodology is reliable, and the numerical tests indicate that the suggested method is highly accurate and requires less computational tools than the current methods. Other than its numerical performance, the technique aids in real world simulation like the transportation of pollutants, heat conduction, and wave propagation in non-homogeneous media. The results highlight the subdivision collocation method as a promising tool for efficiently solving fractional partial differential equations while contributing to global sustainability challenges.

## Introduction

The advection–diffusion equation (ADE) is a fundamental model used to simulate natural processes. It has two main components. Advection describes the transport of substances caused by bulk motion from one region to another. Diffusion represents the movement of substances from areas of higher to lower concentration. This model is widely applied in both natural sciences and engineering. Examples include air and river water transport, pollutant adsorption in soils, and food processing. It is also used in biological system modeling, finance, electromagnetism, fluid mechanics, structural dynamics, and quantum physics. The fractional advection–diffusion equation is well suited for modeling complex transport phenomena. Classical integer-order models often fail to capture anomalous behavior, but the fractional version addresses this limitation. It is widely used in hydrology to describe pollutant dispersion and groundwater contamination, where solute transport often shows heavy-tailed distributions and memory effects. In petroleum engineering, it models fluid flow in porous reservoirs with heterogeneous structures. In biology and medicine, it describes anomalous diffusion of nutrients, drugs, and particles in tissues and blood flow. In physics, it is applied to plasma transport, viscoelastic materials, and charge carrier transport in disordered media. Its applicability to pollutant transport, anomalous heat conduction, and wave propagation highlights its practical value. In finance, similar formulations are used to capture memory-driven price fluctuations and option pricing under anomalous diffusion. These diverse applications highlight the equation’s ability to capture non-locality and long-range dependence, making it more realistic than classical models for many natural and engineered systems.

Recent literature on the time fractional advection-diffusion equation (TFADE) reflects significant advances in both theoretical development and numerical methods, with applications spanning environmental modeling, engineering, and applied mathematics. For instance^1^, investigated pollutant dispersion using a one-dimensional TFADE with the Caputo derivative, demonstrating improved modeling of air pollution dynamics compared to classical models. In^2^ addressed the variable-order TFADE, introducing a spectral collocation method based on shifted Gegenbauer polynomials for efficient numerical approximation, particularly in multi-species transport scenarios. In^3^ proposed a high-accuracy barycentric Lagrange interpolation collocation method for TFADEs, showing that their approach achieves superior convergence and accuracy when compared to existing schemes. In^4^developed a numerical method using operational matrices based on Hosoya polynomials to approximate solutions of TFADEs with Atangana-Baleanu derivatives, converting the problem into a set of algebraic equations and providing error bounds to validate their method’s accuracy. In the context of multi-term TFADEs^5^, presented a fast difference scheme, which significantly reduces computational cost while maintaining accuracy for complex fractional models. For two-dimensional problems, a variable-step high-order finite difference method has been proposed by^6^, enhancing the efficiency and precision of numerical solutions for 2D TFADEs. In^7^ analyzed both analytical and numerical solutions for non-autonomous time-fractional advection-diffusion-reaction equations, underlining the importance of fractional models in capturing complex transport-reaction dynamics. In^8^ introduced a reliable numerical approach for approximating TFADE solutions, confirming the method’s effectiveness through comparative studies. In^9^ presented a novel approach for multi-term TFADEs, utilizing Lagrange squared interpolation for temporal discretization and shifted Legendre polynomials for spatial discretization, with demonstrated for two-dimensional TFADEs under the Caputo derivative, leveraging barycentric rational interpolation and providing rigorous error analysis, thus contributing to the development of robust and precise computational tools for fractional transport equations. In^10^ determined the numerical solution of high dimensional variable-order time fractional diffusion equation via the singular boundary method. These recent studies highlight the ongoing evolution of analytical and numerical techniques for TFADEs, driven by the need to more accurately represent memory effects and anomalous diffusion in real-world systems.

Recently, the subdivision collocation algorithm has emerged as an effective technique. It has been applied successfully to both linear and nonlinear equations. This approach was first introduced by^11^, who applied an interpolatory subdivision technique to develop numerical differentiation and integration formulas. Initially, these methods were designed to generate smooth curves. Subsequently^12–14^, extended these algorithms to address higher-order linear and nonlinear boundary value problems. More recently, the subdivision collocation method has been employed to solve various well-known partial differential equations, as illustrated in studies^15,16^. However, despite these advancements, the time fractional Advection diffusion equation has yet to be addressed numerically using the subdivision collocation method. This work is the first application of subdivision collocation techniques for time-fractional advection–diffusion models with constant and variable coefficients. In contrast to existing collocation or spline-based schemes, the proposed approach makes use of subdivision-generated smooth basis functions that provide improved numerical stability and accuracy for fractional-order problems. In addition, the proposed method attains reliable accuracy with relatively low computational cost as compared to the existing approaches in literature, making it particularly effective for long-time simulations of fractional advection diffusion phenomena. Consider the time fractional advection diffusion equation

1 $$\begin{aligned} \frac{\partial ^\alpha R(x,t)}{\partial t^\alpha } + \beta (x,t) \frac{\partial R(x,t)}{\partial x} + \gamma (x,t) \frac{\partial ^2 R(x,t)}{\partial x^2} = f(x,t), \quad t> 0, \; x \in \Omega , \; 0 < \alpha \le 1, \end{aligned}$$

where $\beta (x,t)$, $\gamma (x,t)$ are variable coefficients. We restrict to a bounded domain $\Omega = [a,b]$ and assume bounded initial condition $R(x,0) = \phi (x)$ and Dirichlet boundary conditions $R(a,t) = g_0(t)$ and $R(b,t) = g_1(t)$ for all $t \ge 0$.

The time fractional derivative in (1) uses the Caputo fractional partial derivative of order $\alpha$, defined as:

2 $$\begin{aligned} \frac{\partial ^\alpha R(x,t)}{\partial t^\alpha } = \frac{1}{\Gamma (1-\alpha )} \int _0^t \frac{\partial R(x,s)}{\partial t} (t-s)^{-\alpha } \, ds, \quad 0 < \alpha \le 1, \end{aligned}$$

where $\Gamma (\cdot )$ is the Gamma function.

The paper is structured in the following way: Section "Derivation of Numerical Scheme’ will give the Derivation of numerical scheme. The initial state of the proposed iterative scheme is available in Section “Initial State”. These sections “Stability” and “Error Estimation” contain an analysis of stability and error estimation outcomes of the current technique respectively. Section "Numerical Results and Discussion" will contain the numerical findings when using the current method and compare them with other methods. Finally, Section “Conclusion” is a conclusion of all the key findings, implications of these findings, discussing its implications, and closing remarks of the research findings.

**Methodology**

Here, we describe the numerical framework used to solve the time-fractional advection diffusion equation based on a subdivision collocation approach.

- Time-fractional advection-diffusion equation with variable coefficients is considered, where the time derivative is defined in the Caputo sense with $0<\alpha <1$.
- A uniform time step $\Delta t$ is used, and the Caputo fractional derivative is approximated by a finite difference convolution formula. An implicit time-stepping scheme is employed to ensure numerical stability.
- The spatial domain is discretized using a subdivision collocation method with smooth, compactly supported subdivision-generated basis functions.
- The governing equation is enforced at the spatial grid points, resulting in a system of algebraic equations at each time level.
- Boundary conditions are imposed using quintic polynomial interpolation consistent with the reproduction degree of the subdivision scheme to ensure smoothness and uniqueness.
- Analyze the stability of the numerical method to ensure reliable solutions. Evaluate error estimates by comparing numerical results with benchmark solutions.
- The approximated recursive equation will be computed by using a suitable numerical technique.
- Finally, we assess the convergence rate, and present the results in the form of graphs and tables.

## Derivation of numerical scheme

In this section, to solve the TFADE model (1), the Caputo fractional derivative is used for time discretization, and subdivision basis functions are used for spatial discretization.

### Time fractional derivative approximation

An approximation to the time fractional derivative can be obtained by a simple quadrature formula given as:

$$\frac{\partial ^\alpha R(x_\ell , t_{n+1})}{\partial t^\alpha } = \frac{1}{\Gamma (1-\alpha )} \int _0^{t_{n+1}} \frac{\partial R(x_\ell ,s)}{\partial t} (t_{n+1} - s)^{-\alpha } \, ds$$

$$= \frac{1}{\Gamma (1-\alpha )} \sum _{k=0}^n \int _{k\Delta t}^{(k+1)\delta t} \frac{\partial R(x_\ell ,s)}{\partial t} (t_{n+1} - s)^{-\alpha } \, ds$$

Using forward difference scheme equation can be reshaped as:

3 $$\begin{aligned} = \frac{(\Delta t)^{-\alpha }}{\Gamma (2-\alpha )} \sum _{k=0}^n \left[ R(x_\ell )^{n+1-k} - R(x_\ell )^{n-k} \right] \left[ (k+1)^{1-\alpha } - k^{1-\alpha } \right] + \mathcal {O}((\Delta t)^{2-\alpha }), \end{aligned}$$

shifting the indices we get,

4 $$\begin{aligned} = a_\alpha \left[ R(x_\ell )^{n+1} - R(x_\ell )^n \right] + a_\alpha \sum _{k=1}^n b_\alpha (k) \left[ R(x_\ell )^{n+1-k} - R(x_\ell )^{n-k} \right] + \mathcal {O}((\Delta t)^{2-\alpha }), \end{aligned}$$

where

5 $$\begin{aligned} a_\alpha = \frac{(\Delta t)^{-\alpha }}{\Gamma (2-\alpha )}, \quad b_\alpha (k) = (k+1)^{1-\alpha } - k^{1-\alpha }. \end{aligned}$$

Now using (4) and theta weighted in (1) we get

6 $$\begin{aligned} a_\alpha R_\ell ^{n+1}+ \theta \gamma _\ell ^{n+1}(R_{xx})_\ell ^{n+1}= a_\alpha R_\ell ^n - a_\alpha \sum _{k=1}^n b_\alpha (k) \big (R_\ell ^{n+1-k} - R_\ell ^{n-k}\big )-\beta _\ell ^{n+1}(R_x)_\ell ^n - (1-\theta ) \gamma _\ell ^{n+1} (R_{xx})_\ell ^n+f^n(x_\ell , t). \end{aligned}$$

### Subdivision scheme

Subdivision schemes are efficient methods for constructing smooth curves or surfaces from discrete data through infinite refinement. This section presents the fundamental concepts of subdivision schemes required to address the proposed problem.

The following 6-point binary interpolatory subdivision scheme (BISS) proposed by^11^ takes a polygon as input and generates a refined polygon as output. To obtain the refined polygon, the 6-point BISS uses six consecutive points of the coarse polygon to compute one new point corresponding to each edge, while retaining the original vertices of the coarse polygon.

7 $$\begin{aligned} \left\{ \begin{array}{l} Q^{k+1}_{2i}=Q_i^k\\ Q^{k+1}_{2i+1}=\frac{3}{256}\left( Q_{i-2}^{k}+Q_{i+3}^{k}\right) -\frac{25}{256}\left( Q_{i-1}^{k}+Q_{i+2}^{k}\right) +\frac{75}{128}\left( Q_i^{k}+Q_{i+1}^{k}\right) \\ \end{array}\right. \end{aligned}$$

Here, $Q_i^k$ and $Q_i^k$ represents the control points of the polygon at the $k^{th}$ and $(k+1)^{th}$ levels respectively. Some properties of this schemes are in the following;

i. The scheme in (7) is $C^2$ continuous.
ii. It has support width (−5,5).
iii. Approximation order of this scheme is six.
iv. The fundamental solution of (7) is defined as
  8 $$\begin{aligned} \rho (x)=\sum \limits _{q\in Z}d_pp(2x-q) \end{aligned}$$
  Moreover, (8) must the satisfy basis limit functions as defined in (9)
  9 $$\begin{aligned} \rho (x)=\left\{ \begin{array}{l} 1 \qquad x=0, \\ 0 \qquad x\ne \ 0. \\ \end{array}\right. \end{aligned}$$

The derivatives of (9) are tabulated in the Table 1.

**Table 1:** Values of basis functions.

*x*	0	$\pm 1$	$\pm 2$	$\pm 3$	$\pm 4$
$\rho$	1	0	0	0	0
$\rho '$	0	$\pm \frac{544}{365}$	${\mp }{\frac{106}{365}}$	$\pm \frac{32}{1095}$	$\pm \frac{1}{1460}$
$\rho ''$	$-\frac{295}{28}$	$\frac{712}{105}$	$-\frac{184}{105}$	$\frac{8}{35}$	$\frac{3}{280}$

### Subdivision collocation method

Let, *N* be a positive integer, where $N\ge 4$ and ${h = {(b-a)/N}}$, $x_\ell =a+\ell h$, where $0\le \ell \le N$. The approximate solution of *R*(*x*, *t*) can be expressed as

10 $$\begin{aligned} R(x_\ell , t_n) = \sum _{q=\ell -4}^{\ell +4} \frac{1}{\xi _{\ell -q}}c_q^n \rho \bigg (\frac{x_\ell - x_q}{h}\bigg )^{w} \end{aligned}$$

where

11 $$\begin{aligned} \xi _{\ell -q}=\left\{ \begin{array}{l} 1 \qquad \ell \ne q, \\ 0<\xi _0<1 \qquad \ell =q. \\ \end{array}\right. \end{aligned}$$

this implies

12 $$\begin{aligned} R^n_{\ell }(x) = \frac{1}{\xi _0}c^{n}_\ell , \,\ \text{ and } \,\ R^{n+1}_{\ell }(x)= & \frac{1}{\xi _0}c^{n+1}_\ell . \end{aligned}$$

Using (12), (10) and its derivatives in (6):

13 $$\begin{aligned} & \frac{a_\alpha }{\xi _0}c_\ell ^{n+1}+ \theta \gamma _\ell ^{n+1}\bigg [\frac{w^2}{h^2}\sum _{q=\ell -4}^{\ell +4} \frac{1}{\xi _{\ell -q}}c_q^{n+1} \rho ''\bigg (\frac{x_\ell - x_q}{h}\bigg )^w\bigg (\frac{x_\ell - x_q}{h}\bigg )^{2(w-1)}+\frac{w(w-1)}{h^2} \sum _{q=\ell -4}^{\ell +4} \frac{1}{\xi _{\ell -q}}c_q^{n+1} \nonumber \\ & \rho '\bigg (\frac{x_\ell - x_q}{h}\bigg )^{w}\bigg (\frac{x_\ell - x_q}{h}\bigg )^{w-2}\bigg ]= \frac{a_\alpha }{\xi _0}c_\ell ^{n}- a_\alpha \sum _{k=1}^n b_\alpha (k) \bigg (\frac{c_\ell ^{n+1-k}}{\xi _0}- \frac{c_\ell ^{n-k}}{\xi _0}\bigg )-\beta _\ell ^{n+1}\bigg [\frac{w}{h}\sum _{q=\ell -4}^{\ell +4} \frac{1}{\xi _{\ell -q}}c_q^n \nonumber \\ & \rho '\bigg (\frac{x_\ell - x_q}{h}\bigg )^{w}\bigg (\frac{x_\ell - x_q}{h}\bigg )^{w-1}\bigg ] - (1-\theta )\gamma _\ell ^{n+1} \bigg [\frac{w^2}{h^2}\sum _{q=\ell -4}^{\ell +4} \frac{1}{\xi _{\ell -q}}c_q^n \rho ''\bigg (\frac{x_\ell - x_q}{h}\bigg )^w\bigg (\frac{x_\ell - x_q}{h}\bigg )^{2(w-1)}\nonumber \\ & +\frac{w(w-1)}{h^2}\sum _{q=\ell -4}^{\ell +4} \frac{1}{\xi _{\ell -q}}c_q^n \rho '\bigg (\frac{x_\ell - x_q}{h}\bigg )^{w}\bigg (\frac{x_\ell - x_q}{h}\bigg )^{w-2}\bigg ]+f^n(x_\ell , t). \end{aligned}$$

As $x_\ell =\ell h$ and $x_q=qh$ (13) becomes

14 $$\begin{aligned} & \frac{a_\alpha }{\xi _0}c_\ell ^{n+1}+ \sum _{q=\ell -4}^{\ell +4} \frac{1}{\xi _{\ell -q}} c_q^{n+1}\bigg [\theta \gamma _\ell ^{n+1} \bigg (\frac{w^2}{h^2} \rho ''(\ell -q)^w(\ell -q)^{2(w-1)}+\frac{w(w-1)}{h^2} \nonumber \\ & \rho '(\ell -q)^{w}(\ell -q)^{w-2}\bigg )\bigg ]= \frac{a_\alpha }{\xi _0}c_\ell ^{n}- a_\alpha \sum _{k=1}^n b_\alpha (k) \bigg (\frac{c_\ell ^{n+1-k}}{\xi _0}- \frac{c_\ell ^{n-k}}{\xi _0}\bigg )-\sum _{q=\ell -4}^{\ell +4} \frac{1}{\xi _{\ell -q}} c_q^{n}\bigg [\beta _\ell ^{n+1}\bigg (\frac{w}{h} \nonumber \\ & \rho '(\ell -q)^{w}(\ell -q)^{w-1}\bigg ) - (1-\theta )\gamma _\ell ^{n+1} \bigg (\frac{w^2}{h^2} \rho ''(\ell -q)^w(\ell -q)^{2(w-1)}\nonumber \\ & +\frac{w(w-1)}{h^2} \rho '(\ell -q)^{w}(\ell -q)^{w-2}\bigg )\bigg ]+f^n(x_\ell , t). \end{aligned}$$

The system of equations (14) can be simplified as

15 $$\begin{aligned}&\frac{a_\alpha }{\xi _0}c_\ell ^{n+1}+ \sum _{q=\ell -4}^{\ell +4} \frac{1}{\xi _{\ell -q}}c_q^{n+1}A_q= \frac{a_\alpha }{\xi _0}c_\ell ^{n} - \frac{a_\alpha }{\xi _0} \sum \limits _{k=1}^n b_\alpha (k) \big (c_\ell ^{n+1-k} - c_\ell ^{n-k}\big )- \sum _{q=\ell -4}^{i+4} \frac{1}{\xi _{\ell -q}}c_q^{n}B_q+f_\ell ^n. \end{aligned}$$

with $A_q$ and $B_{q}$ are defined in ().

$$\begin{aligned} \left\{ \begin{array}{l} A_q =\theta \gamma _\ell ^{n+1} \bigg (\frac{w^2}{h^2} \rho ''(\ell -q)^w(\ell -q)^{2(w-1)}+\frac{w(w-1)}{h^2} \rho '(\ell -q)^{w}(\ell -q)^{w-2}\bigg )\\ B_q =\beta _\ell ^{n+1}\bigg (\frac{w}{h}\rho '(\ell -q)^{w}(\ell -q)^{w-1}\bigg ) - (1-\theta )\gamma _\ell ^{n+1} \bigg (\frac{w^2}{h^2}\rho ''(\ell -q)^w(\ell -q)^{2(w-1)}\\ +\frac{w(w-1)}{h^2} \rho '(\ell -q)^{w}(\ell -q)^{w-2}\bigg ) \end{array} \right. \end{aligned}$$

The matrix will become like

16 $$\begin{aligned} \mathbb AC^{n+1}=\mathbb BC^{n}+\mathbb D- \frac{a_\alpha }{\xi _0}\sum \limits _{k=1}^n b_\alpha (k) \big (c_\ell ^{n+1-k} - c_\ell ^{n-k}\big )\qquad \qquad , \end{aligned}$$

where $\mathbb A$, $\mathbb B$ $C^{n+1}$ $C^{n}$ are defined in (17)-(20).

17 $$\begin{aligned} \mathbf {\mathbb A}=\left( \begin{array}{cccccccccccccc} A_{-4} & ....& \big (\frac{A_{0}}{\xi _0}+\frac{a_\alpha }{\xi _0} \big )& A_{1} & ...& 0 & 0\\ 0 & ....& A_{0} & \big (\frac{A_{1}}{\xi _0}+\frac{a_\alpha }{\xi _0} \big )& ...& 0 & 0\\ 0 & ....& A_{0} & A_{1} & ... & 0 & 0\\ \vdots \\ 0 & ...& 0 & 0 & ...& A_{N+4} & 0\\ 0 & ...& A_{N-1} & \big (\frac{A_{N}}{\xi _0}+\frac{a_\alpha }{\xi _0} \big ) & ...& A_{N+3} & A_{N+4}\\ \end{array} \right) _{(N+1)\times (N+9)}. & \end{aligned}$$

18 $$\begin{aligned} C^{n+1}=(c^{n+1}_{-4},c^{n+1}_{-3},\cdots , c^{n+1}_{N+4}). \end{aligned}$$

19 $$\begin{aligned} \mathbf {\mathbb B}=\left( \begin{array}{cccccccccccccc} B_{-4} & ....& (\frac{a_\alpha }{\xi _0}+\frac{B_{0}}{\xi _0}) & B_{1} & ...& 0 & 0\\ 0 & ....& B_{0} & (\frac{a_\alpha }{\xi _0}+\frac{B_{1}}{\xi _0}) & ...& 0 & 0\\ 0 & ....& B_{0} & B_{1} & ... & 0 & 0\\ \vdots \\ 0 & ...& 0 & 0 & ...& B_{N+4} & 0\\ 0 & ...& B_{N-1} & (\frac{a_\alpha }{\xi _0}+\frac{B_{N}}{\xi _0}) & ...& B_{N+3} & B_{N+4}\\ \end{array} \right) _{(N+1)\times (N+9)} & \end{aligned}$$

20 $$\begin{aligned} C^n=(c^{n}_{-4},c^{n}_{-3},\cdots c^{n}_{N+4})^{T}. \end{aligned}$$

21 $$\begin{aligned} \mathbf {\mathbb D}=\left( \begin{array}{c} f^n_0 \\ f^n_1 \\ f^n_2 \\ \vdots \\ f^n_{N-1} \\ f^n_N \\ \end{array} \right) _{(N+1)\times 1} & \end{aligned}$$

system in (16) can be categorized in form of two cases:

**Case 1 for** n=0

22 $$\begin{aligned} \mathbb AC^{n+1}=\mathbb BC^{n}+\mathbb D \end{aligned}$$

**Case 2 for** $$n\ge 1$$

23 $$\begin{aligned} \mathbb AC^{n+1}=\mathbb BC^{n}+\mathbb D- \frac{a_\alpha }{\xi _0} \sum \limits _{k=1}^n b_\alpha (k) \big (c_\ell ^{n+1-k} - c_\ell ^{n-k}\big )\qquad \qquad , \end{aligned}$$

(23) can also be written as

24 $$\begin{aligned} \mathbb AC^{n+1}=\mathbb BC^{n}+\mathbb D-\mathbb G \end{aligned}$$

25 $$\begin{aligned} \mathbf {\mathbb G}=\left( \begin{array}{c} \frac{a_\alpha }{\xi _0} \sum \limits _{k=1}^n b_\alpha (k) \big (c_0^{n+1-k} - c_0^{n-k}\big ) \\ \frac{a_\alpha }{\xi _0} \sum \limits _{k=1}^n b_\alpha (k) \big (c_1^{n+1-k} - c_1^{n-k}\big ) \\ \frac{a_\alpha }{\xi _0} \sum \limits _{k=1}^n b_\alpha (k) \big (c_2^{n+1-k} - c_2^{n-k}\big ) \\ \vdots \\ \frac{a_\alpha }{\xi _0} \sum \limits _{k=1}^n b_\alpha (k) \big (c_{N-1}^{n+1-k} - c_{N-1}^{n-k}\big ) \\ \frac{a_\alpha }{\xi _0} \sum \limits _{k=1}^n b_\alpha (k) \big (c_N^{n+1-k} - c_N^{n-k}\big ) \\ \end{array} \right) _{(N+1)\times 1} & \end{aligned}$$

### Boundary treatment

Since the matrix $\mathbb A$ and $\mathbb B$ has ${(N+1)\times (N+9)}$ order so in order to get unique solution we have to use the given boundary conditions.

26 $$\begin{aligned} R(x_0,t)=g_0(t). \end{aligned}$$

27 $$\begin{aligned} R(x_N,t)=g_1(t). \end{aligned}$$

However, by adding these two given conditions to the system (16) the systems remains still unstable. So we have to add six more for the determination of unknowns and to obtain a stable system.

### Necessitated conditions

The additional closure conditions are required because the subdivision collocation discretization leads to an underdetermined linear system due to the extended support of the six-point interpolating subdivision basis. These conditions are derived from quintic polynomial interpolation, consistent with the scheme’s degree-five polynomial reproduction, and play a role analogous to end conditions in classical spline theory and boundary rules in subdivision schemes. Different boundary closure strategies may be employed; however, those boundary closure should be selected that preserve polynomial reproduction prevent loss of accuracy near the boundary and maintain the stability and convergence rate of the method. The numerical results and stability analysis confirm that the proposed closure conditions yield a stable, high-order method. We have

28 $$\begin{aligned} R_{-v}{:}{=}S_1(-x_v), \qquad v=1,2,3, \end{aligned}$$

$$\begin{aligned} S_1(-x_v)=\sum \limits _{k=0}^{6}\left( \begin{array}{c}6\\ k\end{array}\right) (-1)^{k+1} R_{-v+k}. \end{aligned}$$

As $$R_v=c_v$$

So (28) will become as:

29 $$\begin{aligned} c_{-v}=\sum \limits _{k=1}^{6}\left( \begin{array}{c}6\\ k\end{array}\right) (-1)^{k+1} c_{-v+k} \qquad v=1,2,3, \end{aligned}$$

Now, for $v=1$, (29) will become

$$\begin{aligned} c_{-1}=\sum \limits _{k=1}^{6}\left( \begin{array}{c}6\\ k\end{array}\right) (-1)^{k+1} c_{-1+k}. \end{aligned}$$

After expanding above expression, we get

$$\begin{aligned} & c_{-1}=\left( \begin{array}{c}6\\ 1\end{array}\right) (-1)^2 c_{0}+\left( \begin{array}{c}6\\ 2\end{array}\right) (-1)^3 c_{1}+\left( \begin{array}{c}6\\ 3\end{array}\right) (-1)^4 c_{2}+\left( \begin{array}{c}6\\ 4\end{array}\right) (-1)^5 c_{3}+ \nonumber \\ & \qquad \qquad \qquad \left( \begin{array}{c}6\\ 5\end{array}\right) (-1)^6 c_{4}+\left( \begin{array}{c}6\\ 6\end{array}\right) (-1)^7 c_{5}. \end{aligned}$$

30 $$\begin{aligned} \Rightarrow c_{-1}-6c_{0}+15c_1-20c_2+15c_3-6c_4+c_5=0. \end{aligned}$$

Now, for $v=2$, (29) will become

$$\begin{aligned} c_{-2}=\sum \limits _{k=1}^{6}\left( \begin{array}{c}6\\ k\end{array}\right) (-1)^{k+1} c_{-2+k}. \end{aligned}$$

After expanding above expression, we get

$$\begin{aligned} & c_{-2}=\left( \begin{array}{c}6\\ 1\end{array}\right) (-1)^2 c_{-1}+\left( \begin{array}{c}6\\ 2\end{array}\right) (-1)^3 c_{0}+\left( \begin{array}{c}6\\ 3\end{array}\right) (-1)^4 c_{1}+\left( \begin{array}{c}6\\ 4\end{array}\right) (-1)^5 c_{2}+ \nonumber \\ & \qquad \qquad \qquad \left( \begin{array}{c}6\\ 5\end{array}\right) (-1)^6 c_{3}+\left( \begin{array}{c}6\\ 6\end{array}\right) (-1)^7 c_{4}. \end{aligned}$$

31 $$\begin{aligned} \Rightarrow c_{-2}-6c_{-1}+15c_0-20c_1+15c_2-6c_3+c_4=0. \end{aligned}$$

Now, for $v=3$, (29) will become

$$\begin{aligned} c_{-3}=\sum \limits _{k=1}^{6}\left( \begin{array}{c}6\\ k\end{array}\right) (-1)^{k+1} c_{-3+k}, \end{aligned}$$

after expanding above expression, we get

$$\begin{aligned} & c_{-3}=\left( \begin{array}{c}6\\ 1\end{array}\right) (-1)^2 c_{-2}+\left( \begin{array}{c}6\\ 2\end{array}\right) (-1)^3 c_{-1}+\left( \begin{array}{c}6\\ 3\end{array}\right) (-1)^4 c_{0}+\left( \begin{array}{c}6\\ 4\end{array}\right) (-1)^5 c_{1}+ \nonumber \\ & \qquad \qquad \qquad \left( \begin{array}{c}6\\ 5\end{array}\right) (-1)^6 c_{2}+\left( \begin{array}{c}6\\ 6\end{array}\right) (-1)^7 c_{3}. \end{aligned}$$

32 $$\begin{aligned} \Rightarrow c_{-3}-6c_{-2}+15c_{-1}-20c_0+15c_1-6c_2+c_3=0. \end{aligned}$$

So, by the equations (30), (31) and (32) the following boundary conditions are employed at left end

33 $$\begin{aligned} \left\{ \begin{array}{l} c_{-3}-6c_{-2}+15c_{-1}-20c_0+15c_1-6c_2+c_3=0 ,\\ c_{-2}-6c_{-1}+15c_0-20c_1+15c_2-6c_3+c_4=0 ,\\ c_{-1}-6c_{0}+15c_1-20c_2+15c_3-6c_4+c_5=0. \end{array}\right. \end{aligned}$$

we obtain a matrix of order $4\times (N+9)$,

$$\begin{aligned} \textbf{L}=\left( \begin{array}{cccccccccccccccccc} 0& 1& -6& 15& -20& 15& -6& 1& 0& 0& \cdots & \cdots & 0& 0& 0\\ 0& 0& 1& -6& 15& -20& 15& -6& 1& 0& 0& \cdots & \cdots & 0& 0\\ 0& 0& 0& 1& -6& 15& -20& 15& -6& 1& 0& 0& \cdots & \cdots & 0\\ 0& 0& 0& 0& 1& 0& 0& 0& 0& 0& \cdots & \cdots & 0& 0& 0\\ \end{array} \right) .\end{aligned}$$

In matrix $\textbf{L}$, first three rows is evaluated through necessitated conditions (33) and last one row come from boundary condition (26).

Similarly for the right end

34 $$\begin{aligned} R_{v}{:}{=}S_1(x_v) \qquad where \qquad v=N+1,N+2,N+3, \end{aligned}$$

$$\begin{aligned} \textbf{S}=\left( \begin{array}{cccccccccccccccccc} 0& 0& 0& \cdots & \cdots & 0& 0& 0& 0& 0& 0& 1& 0& 0& 0& 0\\ 0& 0& 0& 0& \cdots & \cdots & 1& -6& 15& -20& 15& -6& 1& 0& 0& 0\\ 0& 0& 0& 0& 0& \cdots & \cdots & 1& -6& 15& -20& 15& -6& 1& 0& 0\\ 0& 0& 0& 0& 0& 0& \cdots & \cdots & 1& -6& 15& -20& 15& -6& 1& 0\\ \end{array} \right) . \end{aligned}$$

In matrix $\textbf{R}$, first row is evaluated from boundary condition (27) and last three rows are calculated through necessitated conditions (34).

35 $$\begin{aligned} H_1C^{n+1}=G_1C^k+\mathbb D \end{aligned}$$

36 $$\begin{aligned} C^{n+1}=(H_1)^{-1}G_1C^k+\mathbb D \end{aligned}$$

which can be written as

37 $$\begin{aligned} C^{n+1}=K_1C^k+\mathbb D \end{aligned}$$

where $H_1=(L^T,\mathbb A^T,S^T)^T$, $D=(0,0,0,0,\mathbb (D-G)^T,0,0,0,0)^T$ and $G_1=(L^T,\mathbb B^T,S^T)^T$, and $K_1=(H_1)^{-1}G_1$.

**Algorithm 1:**
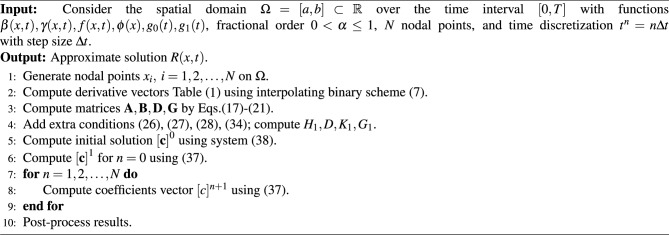
Approximate Solution Procedure.

## Initial state

By using the provided initial conditions i.e.$R(x)=R_0(x)$ and derivative of the initial condition we can determine initial vector. The matrix will be formed like this;

38 $$\begin{aligned} K_1C^0=M_1 \end{aligned}$$

where $$M_1=(0,0,0,g_0(t),\phi _0(x),\phi _1(x),\phi _2(x),\phi _3(x),\cdots , \phi _N(x),g_1(t),0,0,0)^T$$

## Stability

Following the approach in^17^, a Fourier stability analysis is performed for the time-fractional advection–diffusion equation with constant and variable coefficients, demonstrating the unconditional stability of the proposed scheme. Equation (14) can be written as

39 $$\begin{aligned} & \frac{a_\alpha }{\xi _0}c_\ell ^{n+1}+ \sum _{q=\ell -4}^{\ell +4} \frac{1}{\xi _{\ell -q}} c_q^{n+1}\bigg [\theta \gamma _l^{n+1} \bigg (\frac{w^2}{h^2}\rho ''(\ell -q)^w(\ell -q)^{2(w-1)}+\frac{w(w-1)}{h^2} \nonumber \\ & \rho '(\ell -q)^{w}(\ell -q)^{w-2}\bigg )\bigg ]= \frac{a_\alpha }{\xi _0}c_\ell ^{n}- a_\alpha \sum _{k=1}^n b_\alpha (k) \bigg (\frac{c_\ell ^{n+1-k}}{\xi _0}- \frac{c_\ell ^{n-k}}{\xi _0}\bigg )-\sum _{q=\ell -4}^{\ell +4} \frac{1}{\xi _{\ell -q}} c_q^{n}\bigg [\beta _l^{n+1}\bigg (\frac{w}{h} \nonumber \\ & \rho '(\ell -q)^{w}(\ell -q)^{w-1}\bigg ) - (1-\theta )\gamma _l^{n+1} \bigg (\frac{w^2}{h^2} \rho ''(\ell -q)^w(\ell -q)^{2(w-1)}\nonumber \\ & +\frac{w(w-1)}{h^2} \rho '(\ell -q)^{w}(\ell -q)^{w-2}\bigg )\bigg ]+f^n(x_\ell , t). \end{aligned}$$

40 $$\begin{aligned} \frac{a_\alpha }{\xi _0}c_\ell ^{\,n+1} + \frac{1}{\xi _0}\sum _{r=-4}^{4}\frac{1}{\xi _{\ell -r}} a_r^{l,n+1}\,c_{\ell -r}^{\,n+1} = \frac{a_\alpha }{\xi _0}c_\ell ^{\,n} - a_\alpha \sum _{k=1}^{n} b_\alpha (k) \left( \frac{c_\ell ^{\,n+1-k}}{\xi _0}-\frac{c_\ell ^{\,n-k}}{\xi _0}\right) - \frac{1}{\xi _0}\sum _{r=-4}^{4}\frac{1}{\xi _{\ell -r}} b_r^{l,n+1}\,c_{\ell -r}^{\,n}. \end{aligned}$$

Insert $c_{\ell -r}^n = \delta _n e^{iv(\ell -r)h} = \delta _n e^{iv\ell h}e^{-ivrh}$ and divide by $e^{iv\ell h}$. Then

41 $$\begin{aligned} \left( \frac{a_\alpha }{\xi _0}+\sum _{r=-4}^{4} \frac{1}{\xi _{\ell -r}}a_r^{l,n+1} e^{-ivrh}\right) \delta _{n+1} = \left( \frac{a_\alpha }{\xi _0}-\frac{1}{\xi _0}\sum _{r=-4}^{4}\frac{1}{\xi _{\ell -r}} b_r^{l,n+1} e^{-ivrh}\right) \delta _n -\frac{a_\alpha }{\xi _0}\sum _{k=1}^{n} b_\alpha (k)\bigl (\delta _{n+1-k}-\delta _{n-k}\bigr ). \end{aligned}$$

This can be written as

42 $$\begin{aligned} \delta _{n+1}=\frac{Q_1}{Q}\delta _n-\frac{1}{Q}\sum _{k=1}^nb_\alpha (k)\bigg (\delta _{n+1-k}-\delta _{n-k}\bigg ) \end{aligned}$$

where $$Q=1+\frac{\xi _0}{a_\alpha }\sum \limits _{r=-4}^{4}\frac{1}{\xi _{\ell -r}} a_r^{l,n+1} cos(rvh)$$

$$Q_1=1-\frac{\xi _0}{a_\alpha }\sum \limits _{r=-4}^{4} \frac{1}{\xi _{\ell -r}}b_r^{l,n+1} cos(rvh)$$

It is observed that $Q\ge 1, Q_1>0$ for every value of *v*.

**Proposition** Suppose that $\delta _n$ is a solution of $(42)$ we attain

43 $$\begin{aligned} |\delta _n|\le |\delta _0|, n=0(1)N.\end{aligned}$$

Mathematical induction is utilized to verify the inequality eq (43) for $n=0$ system (42) becomes

44 $$\begin{aligned} |\delta _1|=\frac{Q_1}{Q}|\delta _0|\le |\delta _0|, \qquad Q\ge 1 \end{aligned}$$

Suppose that the result $|\delta _n|\le |\delta _0|$ is true for $n=0,1,2,...,N-1$ and from (42) we obtain,

45 $$\begin{aligned} |\delta _{n+1}|\le \frac{Q_1}{Q}|\delta _0|-\frac{1}{Q}\sum _{k=1}^nb_\alpha (k)\bigg (|\delta _{n+1-k}|-|\delta _{n-k}|\bigg )\end{aligned}$$

46 $$\begin{aligned} \le \frac{Q_1}{Q}|\delta _0|-\frac{1}{Q}\sum _{k=1}^nb_\alpha (k)\bigg (|\delta _{0}|-|\delta _{0}|\bigg )\qquad \quad \end{aligned}$$

47 $$\begin{aligned} \le \frac{Q_1}{Q}|\delta _0|\le |\delta _0|\qquad \qquad \qquad \qquad \qquad \qquad \end{aligned}$$

Hence the proposed method is unconditionally stable.

## Error estimation

In this section, we represent some error bounds for the proposed method. The efficiency and the estimation in method are measured by different error norms such as Global relative error(GRE), Euclidean($L_2$), maximal($L_\infty$), Root mean square norm (RMS) and absolute error(*E*). These error norms are defined as follows

$$\begin{aligned} GRE=\frac{\sum \limits _{\ell =0}^N|r(x_\ell ,t)-R(x_\ell ,t)|}{\sum \limits _{\ell =0}^Nr(x_\ell ,t)} \end{aligned}$$

48 $$\begin{aligned} \Vert E\Vert = \Vert r(x_\ell ,t)-R(x_\ell ,t)\Vert \end{aligned}$$

49 $$\begin{aligned} L_{\infty }=\max \limits _{0\le \ell \le N}|r(x,t)-R(x,t)|, \end{aligned}$$

50 $$\begin{aligned} RMS= & \sqrt{\frac{\sum \limits ^{N}_{\tilde{i}=1}\Vert R_{\tilde{i}}-r_{\tilde{i}}\Vert ^2}{N}}, \end{aligned}$$

$$\begin{aligned} L_{2}=\bigg (\sum ^{N}_{\ell =0}|r(x_\ell ,t)-R(x_\ell ,t)|^2\bigg )^\frac{1}{2}. \end{aligned}$$

where *r*(*x*, *t*), *R*(*x*, *t*) are exact solutions and numerical solutions respectively.

## Numerical results and discussion

In this section, we consider the problem(1) for a given spatial grid size *N* (step size $h=\frac{b-a}{N}$) and time step $\Delta t$ the subdivision collocation discretization yields a rectangular system (16), which is converted into a square $(N+9)\times (N+9)$ system (37) by enforcing the boundary conditions and adding six closure constraints based on quintic polynomial (28) and (34). Since the method is iterative we first compute the initial coefficient vector $C^{0}$ by solving the linear system (38). Then the resulting linear system is solved using MATLAB. The iterative system (37) is terminated when desired accuracy is achieved.

### Example 6.1

Let $R(x,t)=x^2+t^2$ represent the exact solution of the time-fractional advection–diffusion equation (1.1) defined over the domain [0,1]. In this example the term $\tfrac{\partial ^\alpha R}{\partial t^\alpha }$ ($0<\alpha \le 1$) is the Caputo fractional derivative in time which includes the effects of memory and hereditary processes, this makes the model useful in the study of the anomalous diffusion in nonhomogeneous and viscoelastic materials. The directed movement of particles, waves, or signals through the spatial domain is regulated by the advection term, $\tfrac{\partial R}{\partial x}$ which is a partial derivative of a density, in fluid mechanics (e.g. turbulent flows) and traffic flow models, where density depends on other densities in a nonlinear manner. The diffusion term $-\tfrac{\partial ^2 R}{\partial x^2}$ used in the transport of pollutants in groundwater, heat in complex materials, charge carriers in disordered media. The external forcing or localized generation/absorption phenomena is provided by the source term on the right-hand side, of the form of the constant $\tfrac{2t^{2-\alpha }}{\Gamma (3-\alpha )} + 2x - 2$, depending upon the physical context.

** Constant coefficients problem:**

$$\begin{aligned} \frac{\partial ^\alpha R}{\partial t^\alpha } + \frac{\partial R}{\partial x} - \frac{\partial R}{\partial x^2} = \frac{2t^{2-\alpha }}{\Gamma (3 - \alpha )} + 2x - 2, \end{aligned}$$

which is one dimensional linear inhomogeneous fractional Burger’s equation.

**Fig. 1:**
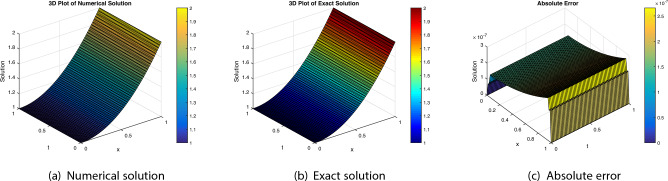
Surface plots for Example (6.1) with $\Delta t=10^{-3}$, $t=1$, $\alpha =0.8$, and $N=60$.

**Fig. 2:**
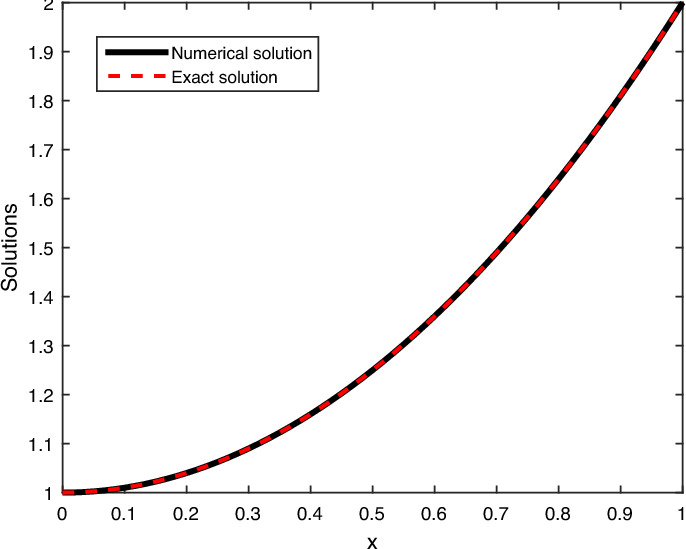
Graph of Example (6.1) for $\Delta t=10^{-3}$,$t=1$, $\alpha =0.8$ and taking $N=60$.

Table 2 presents the accuracy of proposed method through different error norms. The results correspond to $\Delta t = 0.01$, $N = 51$, and $\alpha = 0.5$ over the interval [0, 1] (Table 3). It highlights the superior accuracy of the proposed method compared to the^18,19^ and^20^. Table 4 compares the numerical solution and exact solution at $t=0.0001$. It provides absolute error values for different spatial points when $\Delta t = 10^{-4}$, $N = 410$, and $\alpha = 0.7$. The results demonstrate excellent agreement between the numerical and exact solutions. Table 5 shows GRE, $L_2$, and $L_\infty$ errors at $t=1$ with $\alpha =0.6$. It reports error values for time step sizes ranging from $10^{-2}$ to $10^{-5}$. The data illustrates how reducing $\Delta t$ improves accuracy significantly. Table 3 presents a sensitivity study with respect to the scheme parameter *w*. All other parameters are fixed as $\Delta t = 0.01$, $t = 0.1$, $\theta = 1$, and $\xi _0 = 7.53852185\times 10^{-8}$. The numerical errors are reported in the $L_2$, $L_\infty$, global relative error (GRE), and root mean square (RMS) norms. It is observed that the scheme remains stable for all tested values of *w*. Graphical representation of exact, numerical and absolute errors are represented in Figure 1 and Figure 2.

**Variable coefficients problem:**

$$\begin{aligned} \frac{\partial ^{\alpha } R}{\partial t^{\alpha }} -(x^{2} + t + 1)\frac{\partial ^{2} R}{\partial x^{2}} +t^{2} e^{x} \frac{\partial R}{\partial x} = \frac{2 t^{2-\alpha }}{\Gamma (3 - \alpha )} - 2(x^{2} + t) + 2t^{2}x e^{x} - 2. \end{aligned}$$

Table 6displays the values of $L_{\infty }$, $L_{2}$, and RMS errors computed at $t = 0.2, 0.4, 0.6, 0.8, 1$ with $N = 11$ for $\alpha =0.6$. These results confirm that the proposed method maintains good accuracy for problems involving variable coefficients.

**Table 2:** Error norms corresponding to Example (6.1), for $\Delta t = 0.01$, $N = 51$, $\alpha = 0.5$ in spatial interval [0, 1].

Time	Proposed $L_\infty$	Proposed $L_2$	^18^ $L_\infty$	^18^ $L_2$	^19^ $L_\infty$	^19^ $L_2$
0.1	3.8710e-05	1.3189e-04	6.086e-02	2.613e-01	4.4123e-05	2.2902e-04
0.5	5.0120e-05	1.9848e-04	2.958e-02	1.277e-01	5.1536e-05	2.6637e-04
1.0	8.5774e-05	4.3380e-04	2.114e-02	9.134e-02	5.3363e-05	2.7558e-04
1.5	1.4519e-04	8.4005e-04	1.732e-02	7.485e-02	5.4179e-05	2.7968e-04
2.0	2.2837e-04	1.4000e-03	1.503e-02	6.494e-02	5.4666e-05	2.8213e-04

**Table 3:** Analysis of different errors for Example (6.1), where $\Delta t = 0.01$, $t=0.1$ $\theta =1$, $\xi _0=7.53852e-08$.

Methods	$L_2 error$	$L_\infty$	*GRE*	*RMS*
$w=2$	1.0642e-04	3.1789e-05	3.1990e-05	1.1305e-05
$w=4$	1.3190e-04	3.8710e-05	3.8751e-05	1.3695e-05
$w=6$	1.6745e-04	5.2558e-05	4.8485e-05	1.7135e-05
$w=8$	2.0288e-04	6.3981e-05	2.0744e-05	5.8696e-05
$w=10$	2.1976e-04	6.9306e-05	2.2469e-05	2.2469e-05

**Table 4:** Numerical solution Vs. exact solution of Example (6.1) for $t=0.0001$, $\Delta t=10^{-4}$ and $\alpha =0.7$.

*x*	Exact Solution	Numerical Solution	Absolute Error
0	1e-06	1e-06	0
0.1	0.010001	0.010001	9.074e-12
0.2	0.040001	0.040001	3.8431e-11
0.3	0.090001	0.090001	8.8116e-11
0.4	0.16	0.16	1.5813e-10
0.5	0.25	0.25	2.4847e-10
0.6	0.36	0.36	3.5913e-10
0.7	0.49	0.49	4.9013e-10
0.8	0.64	0.64	6.4145e-10
0.9	0.81	0.81	8.131e-10
1	1	1	0

**Table 5:** The error values corresponding to various time levels for Example (6.1) are calculated at $t = 1$, with $N = 60$ and $\alpha = 0.6$.

Errors	$\Delta t=10^{-2}$	$\Delta t=10^{-3}$	$\Delta t=10^{-4}$
GRE	2.4138e-05	6.0632e-07	1.5230e-08
$L_2$	2.6455e-04	6.6450e-06	1.6692e-07
$L_{\infty }$	4.8479e-05	1.2177e-06	3.0588e-08
*RMS*	3.2789e-05	8.2361e-07	2.0688e-08

**Table 6:** The error results for the variable-coefficient case in Example (6.1) are presented for $\Delta t = 0.01$, $N = 60$.

*t*	$\alpha =0.6$			$\alpha =0.2$		
	$L_{\infty }$	$L_{2}$	RMS	$L_{\infty }$	$L_{2}$	RMS
0.2	$4.8541e-05$	$1.6370e-04$	$1.5177e-05$	$2.7097e-04$	$9.1381e-04$	$8.4720e-05$
0.4	$6.0473e-05$	$2.1736e-04$	$2.2158e-05$	$3.3758e-04$	0.0012	$1.2368e-04$
0.6	$7.8971e-05$	$3.0986e-04$	$3.4258e-05$	$4.4085e-04$	0.0017	$1.9122e-04$
0.8	$1.0530e-04$	$4.5252e-04$	$5.2685e-05$	$5.8786e-04$	0.0025	$2.9407e-04$
1.0	$1.4074e-04$	$6.5555e-04$	$7.8647e-05$	$7.8568e-04$	0.0037	$4.3899e-04$

### Example 6.2

Consider the problem with the exact solution $R(x, t) = t^2 \sin (2\pi x)$. The external forcing term introduces a sinusoidal spatial distribution, while the exact solution $R(x,t)=t^2\sin (2\pi x)$ represents oscillatory spatial behavior with quadratic temporal growth. Physically, such a model can describe heat conduction in composite media, contaminant transport in groundwater, or energy diffusion in viscoelastic materials, making it a meaningful benchmark for testing the accuracy and stability of numerical algorithms.

51 $$\begin{aligned} f(x, t) = \frac{2t^{2 - \alpha } \sin (2\pi x)}{\Gamma (3 - \alpha )} + 4\pi ^2 t^2 \sin (2\pi x), \end{aligned}$$

on [0, 1].

**Fig. 3:**
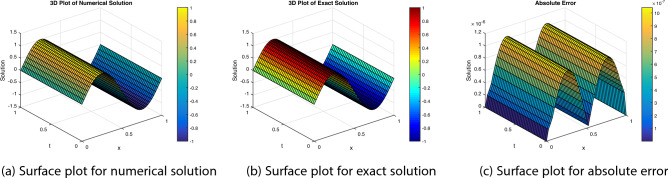
Graph of Example (6.2) for $\Delta t=1.25\times 10^{-3}$, $t=1$, $\alpha =0.6$, and $N=40$.

**Fig. 4:**
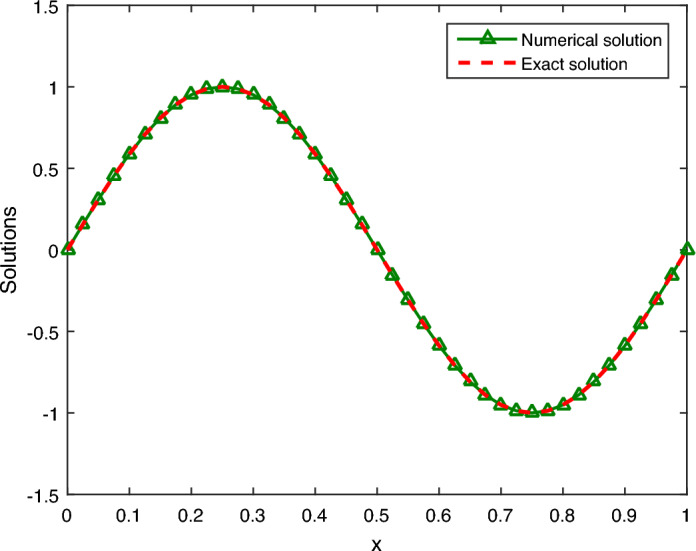
Graph of Example (6.2) for $\Delta t=1.25\times 10^{-3}$, $t=1$, $\alpha =0.6$ and taking $N=40$.

**Fig. 5:**
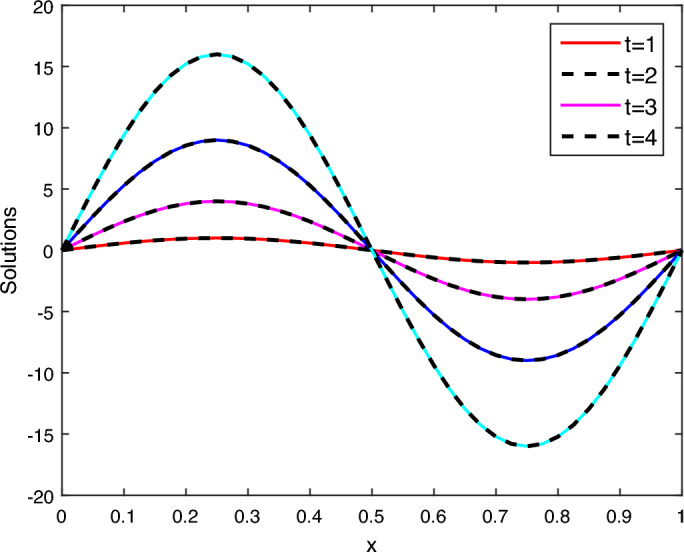
Graph of Example (6.2) for $\Delta t=1.25\times 10^{-3}$, $t=1,2,3,4$, $\alpha =0.6$ and taking $N=40$.

Table 7 compares $L_2$ and $L_\infty$ error norms at $t = 1$ with $\alpha = 0.3$. It presents results from the proposed method alongside those from^20^ and^21^ for various *N* values (Table 8). The data shows that the proposed method consistently achieves smaller errors across all grid sizes. Table 9 demonstrates that the proposed method achieves second-order convergence in both $L_\infty$ and $L_2$ norms while requiring significantly less CPU time than the method reported in^22^, thereby confirming its superior accuracy and computational efficiency. Figure 3 and 4 represents how numerical solution coincides with the exact solution. Figure 5 illustrates the numerical solutions of Example (6.2) at different time levels $t = 1, 2, 3, 4$. Table 8 illustrates the influence of the parameter $\xi _0$ on the accuracy of the proposed subdivision collocation method for Example (6.2). The numerical results are reported in terms of the $L_2$, $L_\infty$, and RMS error norms while keeping $\Delta t = 1.25 \times 10^{-3}$, $t = 1$, $\theta = 1$, $w = 2$, and $N = 5$ fixed. It is observed that all error measures decrease as $\xi _0$ decreases. In particular, reducing $\xi _0$ from $10^{-4}$ to $10^{-7}$ leads to several orders of magnitude reduction in the errors, indicating a significant enhancement in numerical accuracy. This behavior confirms that $\xi _0$ plays a crucial role as a stabilization parameter in the subdivision collocation framework, and an appropriate choice of $\xi _0$ can substantially improve the performance of the method.

The graph shows the variation of solutions over the spatial domain $x \in [0,1]$ for $\alpha = 0.6$. The results confirm good agreement between numerical solutions at different times using $\Delta t = 1.25 \times 10^{-3}$ and $N = 40$.

**Table 7:** A comparison of $L_2, L_\infty$ at $t = 1$ with $\alpha = 0.3$ for Example(6.2).

*N*	^21^		^20^		Proposed Method	
	$L_2$	$L_\infty$	$L_2$	$L_\infty$	$L_2$	$L_\infty$
8	3.4134e−02	4.8273e−02	9.4300e−03	1.5762e−02	3.7784e-05	2.2757e-05
16	8.7334e−03	1.2351e−02	1.1924e−03	2.1670e−03	3.9498e-05	1.3946e-05
32	2.1955e−03	3.1048e−03	1.5040e−04	2.8541e−04	3.4228e-05	8.4937e-06
64	5.4957e−04	7.7721e−04	1.8925e−05	3.6701e−05	2.5671e-05	4.5332e-06
128	1.3739e−04	1.9430e−04	2.3752e−06	4.6559e−06	1.8435e-05	2.3040e-06

**Table 8:** Analysis of different errors for Example (6.2), where $\Delta t = 1.25\times 10^{-3}$, $t=1$, $\theta =1$, $w=2$ and $N=5$.

$\xi _0$	$L_2 error$	$L_\infty$	*RMS*
$2.083e-04$	0.0019	0.0011	7.4322e-04
$4.81e-05$	4.3661e-04	2.4819e-04	1.7044e-04
$9.921e-06$	8.9950e-05	5.1189e-05	3.5117e-05
$3.999e-07$	3.6242e-06	2.0630e-06	1.4150e-06

**Table 9:** A comparison of maximum error $L_2, L_\infty$ at $t = 1$ with $\alpha = 0.3$ and $\Delta t=1.25\times 10^{-3}$ for Example(6.2).

*N*	Proposed Method				^22^			
	$L_\infty$	$L_2$	Order	CPU time	$L_\infty$	$L_2$	Order	CPU time
08	2.8496e-06	4.7312e-06	$\cdots$	0.942	2.2761e-05	5.6903e-06	$\cdots$	6.08404
16	8.7180e-07	2.4692e-06	1.7087	0.998	7.4956e-06	1.3251e-06	1.60246	8.01845
32	2.6543e-07	1.0697e-06	1.7157	0.989	1.7463e-06	2.1829e-07	2.1017	15.3193
64	7.0831e-08	4.0110e-07	1.9059	1.118	1.3761e-07	1.2163e-08	3.6656	34.5386

### Example 6.3

**Fig. 6:**
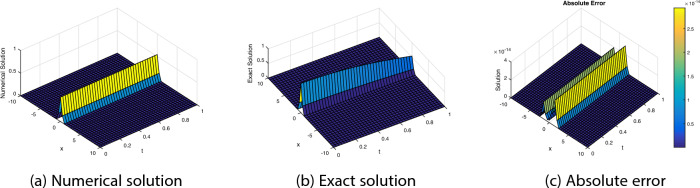
Graph of Example (6.3) for $\Delta t=10^{-5}$, $t=1$, $\alpha =0.8$, and $N=40$.

**Fig. 7:**
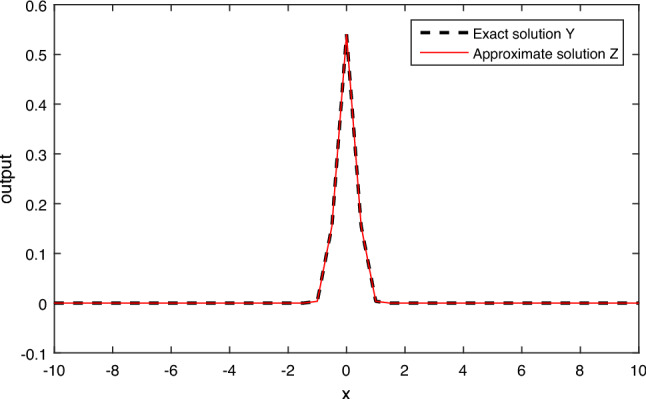
Graph of Example (6.3) for $\Delta t=10^{-5}$, $t=1$, $\alpha =0.8$ and taking $N=40$.

**Fig. 8:**
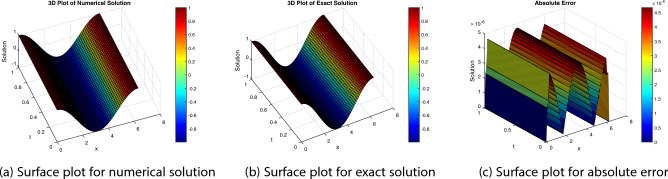
Graph of Example (6.4) for $\Delta t=10^{-2}$, $t=1$, $\alpha =0.6$, and $N=63$.

Consider *ADE* with $\gamma =-1$, $\beta =1$ and the exact solution $R(x,t)=cos(t)\exp ^{-5x^2}$. For $R(x,t)=\cos (t)e^{-5x^2}$ describes a Gaussian profile advected rightward, diffused in space, and oscillating in time under fractional memory effects. Such dynamics arise in applications including oscillatory pollutant plumes, thermal waves in viscoelastic media, and wavepacket transport in fluids or acoustics. The largest absolute errors are reported in Table 10 depending on the values of N. The results are presented in terms of the following parameters: the $\alpha = 0.6$, $\alpha = 0.8$, and $\alpha = 1$, the $\alpha = 1$ in comparison with the proposed method and the results of the refereed article, which is cited in the^23^. In all the cases that are tested, the proposed method results in errors that are much smaller (Figs. 6, 7 and 8).

**Table 10:** Maximum absolute errors in Example (6.3).

*N*	^23^			$Proposed \qquad method$		
	$\alpha =0.6$	$\alpha =0.8$	$\alpha =1$	$\alpha =0.6$	$\alpha =0.8$	$\alpha =1$
10	1.2273e-02	3.7324e-03	2.6389e-03	9.0113e-13	9.9194e-14	1.1367e-14
15	8.8092e-06	9.5638e-06	1.0428e-05	8.7498e-14	9.6316e-15	1.1037e-15
20	2.0316e-07	1.8145e-07	1.5476e-07	4.7874e-13	5.2698e-14	6.0388e-15
25	1.4968e-07	1.5026e-07	1.5194e-07	2.2389e-13	2.4645e-14	2.8241e-15
30	1.1142e-09	1.1733e-09	1.2831e-09	3.3469e-13	3.6842e-14	4.2218e-15

### Example 6.4

Consider the problem with exact solution $R(x,t)=cos(t)t^{\alpha +3}$ on the spatial domain $[0,2\pi ]$. The initial and boundary conditions are derived directly from the exact solution.

**Constant coefficients problem:**

52 $$\begin{aligned} \frac{\partial ^{\alpha } R}{\partial t^{\alpha }} - 10 \frac{\partial ^2 R}{\partial x^2} + 4 \frac{\partial R}{\partial x} = \frac{\Gamma (4+\alpha )}{6} \, t^3 \cos (x) + t^{\alpha +3} \left( 10\cos (x) - 4\sin (x) \right) . \end{aligned}$$

It models a transport that has a memory: Caputo fractional derivative is time nonlocal and subdiffusive, the diffusion coefficient is −10, which means the strength of diffusion in space and the speed of advection is $\beta =4$, the speed of the motion in the positive x-direction direction. The right-hand side is the sum of the forcing the manufactured solution (time-polynomial and spatial trigonometric terms) and source/sink or external driving (e.g. periodic inqection/removal). The formulation is applicable in studies of anomalous heat conduction in complex/viscoelastic materials, oscillatory transport of pollutants in porous aquifers with memory effects, and advected damped wavepackets in fluid or acoustics, where diffusion, advection, and fractional time-effects interact. The outcome in numerical and graphical form has been in the form of tables and figures. The various errors are reported in Table (5.6). It is presented as results are provided at both the values of $\alpha = 0.6$ and N = 63, comparing the suggested method with the one proposed in the^19^ and at that point, our proposed method is better than the one proposed by^19^. The proposed solution has much smaller errors in all the experimented instances.

**Variable coefficients problem:**

53 $$\begin{aligned} \frac{\partial ^{\alpha } R}{\partial t^{\alpha }} - (x+1)\frac{\partial ^2 R}{\partial x^2} + t \sin (x)\frac{\partial R}{\partial x} = \frac{\Gamma (\alpha +4)}{6} t^3 \cos (x) + t^{\alpha +3}(x+1)\cos (x) - t^{\alpha +4}\sin ^2(x). \end{aligned}$$

is concise and physically interpretable: the Caputo fractional derivative $\partial _t^\alpha$ ($0<\alpha \le 1$) models temporal memory and anomalous diffusion, the spatially varying diffusion coefficient $x+1$ represents heterogeneity in material or porous structure that alters local spreading, and the time–space dependent advection coefficient $t\sin (x)$ models a temporally evolving flow with spatial modulation (e.g. seasonal or driven forcing). The right-hand side enforces the manufactured solution and acts as external forcing/source terms. Such a formulation is relevant for transport in heterogeneous porous media with time-varying flows, heat/viscoelastic waves in nonuniform materials, and environmentally driven pollutant transport with spatially varying diffusivity and time-dependent advection (Table 11).

Numerical and graphical results has been represented in form of tables and figures. The $L_{\infty }$, $L_2$ errors, and RMS error values are computed at $t = 0.2, \, 0.4, \, 0.6, \, 0.8, \, 1$ with $N = 63$ for $\alpha = 0.2$ are presented in Table 12 which shows that our proposed method produces better results as compared to other methods in literature.

**Table 11:** The error values for Example (6.4) with constant coefficients where $\Delta t = 0.01$, $N=63$ and $\alpha =0.6$.

*t*	Proposed method			^19^		
	$L_{\infty }$-error	$L_{2}$-error	RMS	$L_{\infty }$-error	$L_{2}$-error	RMS
0.2	$1.4256e-08$	$7.5735e-08$	$8.5238e-09$	$2.4075e-05$	$1.0498e-04$	$1.3226e-05$
0.4	$1.7259e-07$	$9.1683e-07$	$1.0319e-07$	$8.4403e-05$	$3.7126e-04$	$4.6774e-05$
0.6	$7.4242e-07$	$3.9438e-06$	$4.4386e-07$	$1.5743e-04$	$6.9046e-04$	$8.6990e-05$
0.8	$2.0906e-06$	$1.1105e-05$	$1.2498e-06$	$2.1727e-04$	$9.7529e-04$	$1.2287e-04$
1.0	$4.6669e-06$	$2.4791e-05$	$2.7901e-06$	$2.4613e-04$	$1.3247e-03$	$1.6689e-04$

**Table 12:** The error results for the variable-coefficient case in Example (6.4) are obtained for $\Delta t = 0.01$, $N = 63$, and $\alpha = 0.2$.

*t*	^19^			*proposedmethod*		
	$L_{\infty }$-error	$L_{2}$-error	RMS	$L_{\infty }$-error	$L_{2}$-error	RMS
0.2	$2.8216e-06$	$1.2826e-05$	$1.6159e-06$	$1.0358e-07$	$3.7976e-07$	$3.9407e-08$
0.4	$1.1715e-05$	$4.9151e-05$	$6.1924e-06$	$9.5165e-07$	$3.4887e-06$	$3.6200e-07$
0.6	$4.6606e-05$	$2.2879e-04$	$2.8825e-05$	$3.4826e-06$	$1.2767e-05$	$1.3247e-06$
0.8	$1.1889e-04$	$6.1401e-04$	$7.7358e-05$	$8.7431e-06$	$3.2051e-05$	$3.3256e-06$
1.0	$2.4441e-04$	$1.2594e-03$	$1.5866e-04$	$1.7854e-05$	$6.5452e-05$	$6.7911e-06$

### Example 6.5

Consider an example for TFADE (1) with constant coefficients $\beta =0,\gamma =-1$ representing low regularity^24^ where the exact solution is $R(x, t) = t^{2+\alpha } \sin (2\pi x)$. The external forcing $f(x,t)=t^2sin(\pi x) \frac{\Gamma (3+\alpha )}{2 + \pi ^2 t^alpha )}$. Table 13 demonstrates that the proposed subdivision collocation method produces small numerical errors, but fails to achieve the optimal convergence rate.

**Table 13:** Maximum error $E_\infty$ and observed rate of convergence of Example (6.5).

*N*	$\alpha =0.3$		$\alpha =0.6$		$\alpha =0.8$	
	$E_\infty$	Convergence order	$E_\infty$	Convergence order	$E_\infty$	Convergence order
10	$1.4049e-07$	–	$2.0462e-08$	–	$5.8430e-09$	–
20	$8.8109e-08$	0.6731	$4.0852e-09$	2.3245	$1.1824e-09$	2.3050
30	$9.6109-09$	5.4645	$1.4261e-09$	2.5956	$3.8966e-09$	−2.9412
50	$4.8945e-07$	−7.6942	$3.3556e-10$	2.8325	$6.5857e-08$	−5.5349
160	$7.2061e-11$	7.5859	$1.0688e-11$	2.9632	$2.497e-01$	−13.0235

## Conclusion

This paper described a subdivision collocation scheme to solve time-fractional advection diffusion equation with variable coefficients. The method, in addition, through the use of the Caputo fractional derivative to discretize time and subdivision scheme to approximate the space, discretized the governing problem to a system of algebraic equations in an efficient way. Theoretical study proved the unconditional stability, and the error estimates proved convergence properties.

Numerous numerical experiments evidenced that the given approach can provide extremely accurate results at a lower cost of computation than the current methods. The scheme was found to be efficient in terms of memory and CPU usage requiring fewer spatial nodes than in other constant- and variable-coefficient methods. In addition, we have explicitly analyzed the mesh refinement impact by reporting error norms and convergence orders for increasing numbers of spatial nodes while keeping the time step fixed. The numerical results demonstrate that both the $L_{\infty }$ and $L_2$ errors decrease monotonically as the mesh is refined, confirming the convergence of the scheme. The method remains stable under mesh refinement and consistently achieves second-order spatial accuracy, with no saturation effects observed for the tested mesh sizes. Moreover, CPU time comparisons show that the proposed method attains this accuracy with significantly lower computational cost as compared to^22^.

It is so robust and accurate that it has been applied to the modeling of a large variety of real world processes where the effects of memory and anomalous diffusion are dominating such as the pollutant transportation in groundwater, the effect of anomalous heat conduction in viscoelastic media, as well as to the wave propagation in complex fluids.

Therefore, the subdivision collocation approach provides a very strong and sound method towards numerical solution of time fractional advection-diffusion equations. Compared to the results of the proposed method, there is an improvement in the results of the use of the method of the references considered to be the best versions of the approach^18–21,23^:. It is possible that future work can be stretched out to multidimensional, multi-term, and nonlinear fractional models to expand its capability in both scientific and engineering applications.

## Data Availability

The data used to support the findings of the study are available within this paper.

## List of symbols

$\alpha$: Fractional derivative order
$$\Gamma$$: Gamma function
$c_q^n$: Time-dependent subdivision coefficients
*N*: Number of spatial subintervals
$$\gamma (x,t)$$: Diffusion coefficient
$$\beta (x,t)$$: Advection velocity
$\theta$: Time-weighting parameter
*t*: Total simulation time
*h*: Spatial discretization step
$\Delta t$: Temporal discretization step
$Q_i^k$: Control point at spatial index *i* and subdivision level *k*
$\rho (x)$: Fundamental basis function of the six-point interpolating subdivision scheme
*R*(*x*, *t*): Numerical solution at grid point $(x_\ell ,t_n)$

## Subscripts/superscripts

$\ell$: Integer indices for spatial discretization
*n*: Integer indices for temporal discretization
C: Caputo operator

## Code availability

The code is available from the corresponding author upon reasonable request.
